# South African student leaders' role experience through social dream drawing: A driver of compassion

**DOI:** 10.3389/fpsyg.2023.1109169

**Published:** 2023-03-22

**Authors:** Neo Pule, John Gibney

**Affiliations:** ^1^Department of Psychology, University of the Free State, Bloemfontein, South Africa; ^2^Managing Differences Ltd. Australia, Melbourne, VIC, Australia

**Keywords:** compassion, transformation, companionate listening, student leadership, social dream drawing

## Abstract

**Introduction:**

Compassion can be viewed as a central gluing agent for the soul. Coupled with companionship toward a unique quality of listening, we call this companionate listening. South African student leaders' role is central to the decolonization and transformation of higher education based on the legacies founding these institutions. As such, a humanized practice that is centered on more emotional or virtue-embodied approaches than the traditional lens has been sought. This lens offers avenues for innovative, creative, and inclusive perspectives that promote compassion, social justice, and democracy.

**Methods:**

To extend existing conceptualizations of compassion, this research uses social dream drawing to attain “companionate listening” as a means of exploring communication as “exquisite” empathy. Through psychoanalytic theory, companionate listening is theorized from observing the value of social dream-drawing research with South African student leaders.

**Results:**

The drawings show how assuming leadership roles re-ignites feelings and dream images of complex political, historical, social, cultural, and psychological South African intersections that emerge during South African student leadership.

**Discussion:**

It is, therefore, concluded that innovative and inclusive research agendas into new horizons of forms of compassion, like social dream drawing in this research, are necessary within South African student leadership. Accordingly, through social dream drawing, compassionate listening facilitates a process of emotional growth toward an integrated self and group. This is because dreams allow the human capacity for connections that open space for compassion, enabling the feeling of relatedness and connection for student leaders that leads to the more impactful transformation of South African institutions.

## Introduction

This article is introduced with a hypothesis. We hypothesize that through the practice of social dreaming, which combines the drawing of dreams in a group context and sharing the associations to the dreams and drawing, a compassionate field is constructed through these interactions along with multiple shared meanings. In this field, compassion can be viewed as a central gluing agent for the soul, which couples with companionship toward a unique quality of listening, which we call companionate listening. Accordingly, this genre of listening promotes new frontiers of innovative and inclusive research agendas. Thus, in this study, we aim to extend the neuroscience lens on compassion in the context of working with student leaders in South African universities. From a neuroscience perspective, compassion mirrors responsiveness to the suffering of another while incorporating the desire to help. According to Bion (1960 cited in Abel-Hirsch, 2019, p. 171), truth and compassion are human senses that go together and are needed if individuals are to satisfy their needs for curiosity and emotional/mental growth.

Neuroscientists view compassion as a response to stressful conditions fostering both relationships and resilience, which are otherwise not included in empathy (Chierchia and Singer, 2017). In fact, behaving in a compassionate manner affects the body quite powerfully toward healing (McGonigal, 2016); while, according to neuroscience, constant states of stress and anxiety reduce the capacity for compassion (Levine, 2021). Neuroscience differentiates between compassion and empathy, stating that empathy only points to sharing the experience of another's emotional state without the prosocial motivation to become involved in their situation (Singer and Bolz, 2013; Ashar et al., 2016). However, in our extension of the neuroscience lens regarding compassion, psychoanalytic theory views empathetic listening as a co-creative space of meaning toward a cohesive or integrated self or group (Akhtar, 2013; Long, 2016). This type of empathetic listening helps to establish a mutuality that we would like to think of as incorporating compassion and companionship. Thus, by utilizing social dreaming, shared associations, and dream drawings, we create a compassionate community or field which, through shared stories and generational traumas, produces healing, shared meaning, and expanded identity development.

In our focus of concern in this study, the drawings show how assuming leadership roles re-ignites feelings and dream images of the anti-apartheid activism of the 1970s. Some of these are actually relevant to immediate family members of student leaders. However, much of the material emanated from the field (Lewin, 1959), created by the student leaders coming together and the intersections between the social, political, historical, and psychological contexts of universities in South Africa. In 2015/16, the #Fallist movement reacted to a perpetuating, segregated, and unchanging system of black and white universities. There were marked differences in resources and amenities across the university system. For example, historically, white universities restricted entry and familiarity largely to black students who now experience difficulty integrating into the institutional culture of the universities (Bazana and Mogotsi, 2017). English and Afrikaans universities were white populated (Ilorah, 2006), while black universities, which were established to serve black students who were barred from enrolling in white-only universities, remain underfunded and under-resourced (Netshakhuma, 2022). In total, 20 years later, students asked what had changed (Swartz et al., 2019). The answer was nothing much, so students marched, demonstrated, and occupied universities, shutting them down (Griffiths, 2019). The statue of Cecil John Rhodes and other white leaders was torn down to chants of “Fees must Fall” and “Rhodes must Fall” (Griffiths, 2019; Swartz et al., 2019).

Post-apartheid, student leaders in South Africa include student representatives (especially Student Representative Councils or “SRCs”), who are elected and perform their role according to the Higher Education Act 101 of 1997 or the more recent National Student Governance Framework (Republic of South Africa, 1997; South African Association of Senior Student Affairs Professionals, 2021). The Act was implemented as a South African higher education redress measure regarding the post-apartheid era in South Africa (and South African higher education) to operationalize the co-governance strategy to include students in university management decision-making. Student clubs, societies, and community development leaders are also regarded as student leaders according to this framework. In addition, South African universities sought to invest in the development of students' graduate attributes and employability through student leadership development. Thus, various leadership programs such as the South African Washington International Program (SAWIP), the University of the Free State Leadership for Change Program, the Thabo Mbeki African Leadership Institute at the University of South Africa (UNISA), the Future Health Leaders Program of the University of Cape Town, and others, became avenues for student leadership development (Getz and Roy, 2013; Pule, 2017). Students who partake in these programs are referred to as student leaders as they represent universities' efforts toward hands-on leadership and skills development that students can apply to universities' extracurricular agendas. Recently, a new form of student leadership emerged through the #Fallist movements such as #Fees Must Fall and #Rhodes Must Fall, which resulted in other protests or activism regarding fees, the university language of instruction, issues of access, and so on. These student leaders are called activists or non-positional leaders and usually lead justice movements or protests; they receive attention from university management and impact university policies without being linked to any formal leadership role within the university itself (Pule and May, 2021).

The South African student leadership landscape may be fragmented in terms of the consolidated definition of the role of student leadership. However, South African student leaders, regardless of their role, commonly contend with the effects of the injustices of the past, including transformation, decolonization, and social justice issues. Thus, South African student leaders can often be misunderstood, which possibly leads to their role being confused, especially against the background of the recent protests that have been labeled as destructive and violent. This can also lead to a misconstruction of the various forms of student leadership that currently exist and, consequently, a misinterpretation of their student leadership experiences. Though the definition of student leadership may be vague, it ultimately has to do with the way student leaders are taking up their membership in the university, which affects them psychologically and emotionally.

The membership of student leaders in the university and its effects on them psychologically and emotionally are linked to their primary tasks (Long, 2006). The primary task is the one thing the organization must do to survive (Lawrence, 2005). The primary task for student leaders involves attending to South African transformation and decolonization imperatives (Pule and May, 2021). As this primary task involves work beyond the university, it places student leaders as important players within the greater society (Bazana and Mogotsi, 2017). We posit that while this group (of student leaders) is working with this primary task, the group also manages anxiety that surfaces during their interactional space, sparked by the group working with its primary task (Bain, 1998) in the context of the field. Through their interactions, they generate emotional communications or affects that have meaning and move people as a field or energetic mass which co-evolves and spreads and ultimately surrounds the individual, dyad, group, or organization. It is non-verbally communicated through projective identification and parallel processes, which should become awareness tools for diagnosing the dynamics of any situation within the organization. Projective identification “refers to a group of fantasies and accompanying object relations having to do with the ridding of self of the unwanted aspects of self; depositing of those unwanted “parts” into another person and finally with the “recovery” of a modified version of what was extruded” (Ogden, 1979, p. 357). Ogden (1979, p. 358) continued to explain that in a schematic manner, projective identification is a process involving the following sequence: first, there is the fantasy of projecting a part of oneself into another person and of that part taking over the person from within; then, there is pressure exerted *via* the interpersonal interaction such that the “recipient” of the projection experiences pressure to think, feel, and behave in a manner congruent with the projection; finally, the projected feelings, after being “psychologically processed” by the recipient, are reinternalized by the projector. As a result, projective identification facilitates the psychological processing of student leaders' fantasized material, or unconscious thoughts and feelings, by using the interactional space as a holding space for relational exchange. On the foundations of Bion's theorizing, this means that projective identification enables communication to receive, understand and metabolize the unconsciously exchanged thoughts and feelings within the mutually beneficial interaction (Robinson, 2005). A parallel process occurs where two social systems, in contact with each other, demonstrate similar “effects, behaviors, and cognitions” (Adelfer et al. cited in Berg and Smith, 1985, p. 31). [SIC]

Five theoretical concepts set the ground for discussing field creation, co-creating realities, and parallel processes. The five concepts are (Lewin, 1959, p. 27–150; Parlett, 1991 p. 71–73): “Organization - meaning comes from looking at the total situation”, Contemporaneity - nothing exists beyond the “here and now”, “Singularity - avoid generalizations and focus on the unique”, “Changing Process - no moment the same, nothing is fixed or static”, and “Possible Relevance - whatever exists, exists only now as flux is basic to experience”. Heavily influenced by the Gestalt School of Psychology, Lewin describes the qualities of researchers as the use of self, presence, present-centeredness, and focusing on “what is” in the “here and now” at the level of feelings, sensations, dreams, fantasies, images, and so forth (Clarkson, 2000, p.1, 5 and 14). Accordingly, the field brings the psychological past, present, and future in one moment, such as in “Contemporaneity” (Lewin, 1959, p. 27).

Suitably, empathy and intuition are methods of gaining quick and deep understanding while working on the primary task. Empathy is used to reach feelings, while intuition is used to get ideas from thinking by picking up on clues that empathy gathers. Moreover, empathy employs the cognitive, perceptual, and affective capabilities leading to intuition and coming before insight. Understood as a communication between one unconscious and another, listening with this type of empathy is the intersubjective co-produced (Sutanto, 2021). Thus, if a group is viewed as an entity or unit (such as in group-as-a-whole) (Long, 2016), then empathetic listening is a co-creative space of meaning, an approach toward a cohesive or integrated self (or group) proposing a sense of psychic coherence or facilitated notions of hospitality, derived from group members listening to each other “empathetically” (Akhtar, 2013). This hospitality leads to an engagement with and understanding of each other (in the group) and the group's surroundings, which psychoanalysts refer to as transference interpretation, making association conceivable (Sutanto, 2021). Consequently, the group can reach a type of homeostatic connection—as a system—such that continuity of being, validation, and harmony with the environment is realized (Akhtar, 2013). This harmony provides a holding affirmation resembling a deep connection and recognition of the perceptive and experiential states of another. As such, empathy in listening helps to establish a mutuality that overlaps with the neuroscientist's conceptualization of compassion.

However, different stories develop different polarities and perspectives. We propose that these polarities need to be understood to develop a shared reality and sense of belonging through compassion and companionship. Our sense of integrating compassion and companionship is about using emotional communication to listen with empathy while meeting the other's needs and having one's own needs met. Therefore, participants are witnessing and experiencing each other's experience—such that through the witnessing, healing occurs. Consequently, innovative and inclusive research agendas are necessary to humanize research and transform institutional cultures toward restorative and collaborative practice.

## Theoretical framework

Using socio-analysis and specifically social dream drawing, experiences of student leaders as role holders in South African universities were explored for deeper understanding. Socio-analysis, as stated by Long (2017), focuses on the study of role even as an element of society, using a combination of psychoanalysis with systems thinking and group relations to understand the psychology and behaviors of people within their social context. The social context is seen as the collection of thoughts, feelings, actions, and processes of people in-context, which are long-term projected aspects of the social environment of relationships. Employing social dream drawing, the socio-analytic understanding of the student leaders' role experiences was explored by sharing dreams through drawing. According to Mersky (2022), who developed the social dream drawing approach, this is a social thinking process that provides a verbal and visual tool to explore social and psychological issues in a group setting. This manner of exploration helped uncover and verbalize rich insights and meanings about student leaders' experiences of their leadership roles through a creative and playfully spirited methodology.

Another piece of a psychoanalytic theory that may be helpful for the reader is how the field is used in psychoanalytic ways of working. According to Katz (2017, p. 113–115), there are three field theories, stories, metaphors, and dreams, each with a different focus. The analytic style of each researcher gives priority to different aspects of the participant's narrative. Each analyst listens to different emergent configurations of movement in their relational interactions. Like valences in basic assumption groups (Bion, 1948), each individual probably prefers a particular field approach and associated form of listening.

The author's preferred approach is the Oneiric Field model, also known as the “Post Bionic Field Model” (Katz, 2017), relying on what the researchers' and participants' body sensations, daydreams, hallucinosis, and reveries reveal and treating the material as if it were a dream. It follows Bion's contention that we dream all the time, even when we are awake but are not aware of it. It also assumes that we will be projected into by others; hence, we can emotionally and psychically pick up their unconscious material and metabolize it for them (sense-making).

## Methodology

The research was conducted across multiple South African universities located in provinces in Gauteng, the Free State, Eastern and Western Cape, Limpopo, and KwaZulu-Natal. The research sites characterize the various types of South African universities in terms of urban and rural, as well as historically white or black universities, and encompassed the diversity of student populations. The participating student leaders belonged to various structures of student leadership, although mostly linked to student representation councils. In total, 11 social dream drawing groups were held, one session per group. Each group consisted of five to seven student leaders. Participant selection occurred through purposive, volunteering sampling; purposive because the research looked for participation from students who held a leadership role in the university. Student leaders participated without coercion and could withdraw their participation at any point during the process.

Ethical approval for the research was obtained from the University of the Free State, General and Human Research Ethics Committee (GHREC). In addition, institutional permission to conduct research at the various institutions was obtained through the respective institutions' ethics committees.

The research used a qualitative approach, focusing on the descriptions of the dreams and accompanying drawings, as well as on the co-construction of the student leadership experiences presented verbally and visually through social dream drawing. The verbal expression included the description of the dream and corresponding drawings, the associations made to the dreams and drawings, as well as the meaning-making pertaining to student leadership experiences based on the emerging associations and hypotheses inspired by linking dreams (and drawings) or associations, one to another (Mersky, 2022). Verbal data were collected by means of a voice recorder and later transcribed. Visual data were indicated by the drawing of the dreams and photographs of the drawings (Mersky, 2012) that were captured; these were kept in an electronic file. Themes were derived from the collected data by studying the transcribed text and photographs. Based on the themes, the researchers reflected on the impact of social dream drawing in relation to student leadership experiences. They noted the significance of how student leaders shared through social dream drawing and their response regarding their experience of participating in social dream drawing. This reflection raised the researchers' understanding of the role of compassion in the student leaders' sharing and their role experience.

## Social dream drawing

Prior to gathering groups of student leaders using social dream drawing, an information sheet was provided to each student leader who had indicated an interest in participating. Student leaders signed a consent form (Leedy and Ormrod, 2014) and sent this to the principal researcher by email. The researcher managed the process by keeping the groups between five and seven student leaders per group. The information sheet explained the process to be followed during social dream drawing, the expectations, and information about the research, including its aims, objectives, and intentions (Leedy and Ormrod, 2014). Student leaders were asked, through the information sheet, to think of a sleep dream they may have had at some point in their lives: It could have been years, weeks, or days before and could relate to their student leadership experiences (Mersky, 2012). They were asked to make a drawing of this dream and bring it to the group meeting (Mersky, 2022).

The word “social” in social dream drawing refers to the application of socio-analysis and the social context as defined earlier. Accordingly, the researchers and participating student leaders refer to “the group” when conducting social dream drawing (Mersky, 2012). In fact, researchers and participating student leaders (the group) work together to co-construct the understanding of student leadership experiences which led to the synthesis relating to compassion and companionate listening. During the group meeting, the researchers are hosts of the social dream drawing and provide information to student leaders about the process to be followed as a guide through the three-part process of social dream drawing. This guidance pertains to keeping the boundaries of the task for each of the three-part processes, including the time allocated for each. The first part is to share their dream. The corresponding drawing is allocated 10–15 min of the allocated hour; the second is making associations to the dream drawing is allocated 20–25 min. Finally, meaning-making derived from the emerging associations occurs for the remainder of the hour (Mersky, 2012). To proceed through each dream drawing exploration for 1 h each, student leaders take turns to share a dream and corresponding drawing, one at a time, although no order is assigned. Student leaders had the opportunity to share when they felt that they were ready to share. This approach toward social dream drawing is favorable due to the democratic and co-productive nature of the method (Mersky, 2012; Manley, 2014).

Once the dream and its drawing were shared by respective student leaders, one at a time, the group (including researchers and participating student leaders) had the opportunity to ask questions, mostly clarifying, about the dream and the drawing. Afterward, the dreamer (i.e., the student leader taking a turn to share their dream and drawing) placed the drawing in the middle of the group circle, symbolically handing the drawing over to become a tool that the group could use to explore student leadership by initially making associations to the drawing. These associations consisted of anything that came to the mind of group members, such as a song, a movie, a saying, a memory or experience, or another dream (Manley, 2014). Associations are allowed to float and can be shared at any time, organically, once the dream drawings are shared to allow a dynamic process of thoughts, feelings, and reactions to emerge organically without much guidance or control from the researchers (Mersky, 2012; Manley, 2014). Thereafter, the group used the associations to make meaning regarding student leadership. To focus on the meaning-making portion of the three-part social dream drawing processes: The researchers, as the hosts of social dream drawing, verbalized the following guiding statement: *now that we have made these associations, what is the meaning that we make about student leadership experience?* The group responded to this by articulating ideas regarding the meaning that they make about student leadership by doing the following: 1. linking one dream drawing to another, 2. linking dream drawings to contributed associations and 3. linking associations one to another, including allowing emerging associations to raise insights regarding student leadership (Mersky, 2022). Researchers have an important role to play in this part of social dream drawings as they can offer a hypothesis that can be further explored by the group to make meaning of student leadership (Mersky, 2012). As more dreams and drawings were shared, meaning made about student leadership built progressively. Once all the dreams were shared, student leaders were asked to reflect and comment on their experience of the social dream drawing experience. This reflection led to the emergence of the topic discussed in this article regarding compassion and companionate listening, where student leaders indicated the innovative and inclusive kind of listening and connection they experienced from social dream drawing.

## Dream drawings of south african student leaders

Dream drawings showed various aspects of student leadership in South African universities. Using systems' thinking, the issues raised from dream drawings pertained to experiences that can be described as personal and individual student leader experience which entailed experiences involving their respective university, issues cutting across universities, as well as in the higher education sector at large. In this way, personal and individual experiences reflected group (i.e., student leadership at respective campuses or as a South African student leader) and organizational (Higher Education nationally in South Africa or as part of the global sector), and vice versa. Some issues raised related to the South African past, especially during talks about social justice, the role of student leaders in university management and decision-making, how student leadership is perceived by student leaders or the general student population, as well as how leaders in South Africa are perceived.

**Figure 1:**
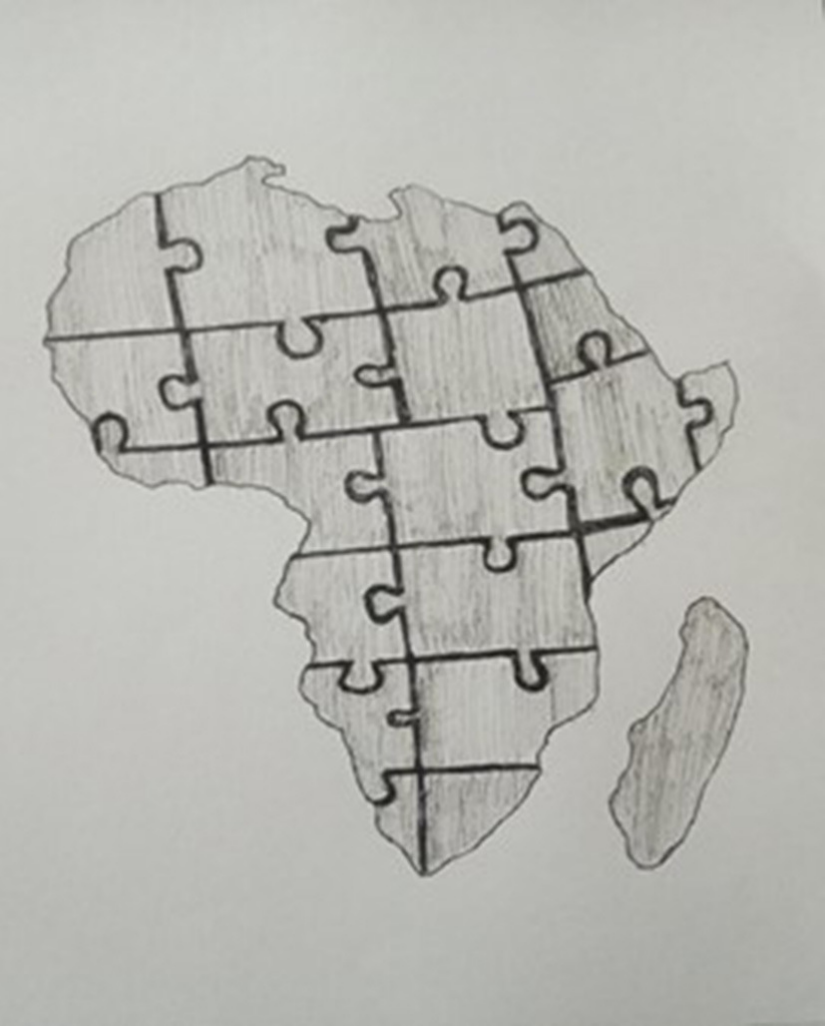
Dream drawing on African unity.

Accordingly, dream drawings showed the significance of unity between student leadership, South African higher education, South Africa, and African leadership. Student leaders felt that unity was an important tool to have for successful student leadership or leadership in general and that unity had a significant role to play in the progress, development, and forward moving of the country. From this, markers of inter- and trans-generational trauma based on the historical–political–social and psychological context and impact of apartheid and post-apartheid emerged. For example, a student leader showed a drawing about a dream that he had about putting together a jigsaw puzzle of the world. In the drawing (Figure 1), the map of Africa is shown.

The hooks holding the jigsaw puzzle together were associated with the nipples of a woman's breast that supplies nourishment and sustenance and denotes care, connection (interconnection), and things being held together. In addition to the role of women in unity that arose in the ensuing conversation, African unity became the primary focus therein. The student leaders wondered whether student leaders were united, at the campus level or on a national level, whether there were possibilities to unite with student leaders across the continent even, and what that might mean for future leadership and the progression and success of Africa. An interesting feature of the drawing was that no hooks (or nipples) were included for South Africa. Across the whole jigsaw puzzle, there were hard and solid lines that boundaried the country from one to another. Therefore, without hooks and with these hard and solid boundaries, South Africa was kept completely removed from the rest of the continent. This implied a unique characterization of South Africa and how student leaders saw the apartheid past and post-apartheid manifestations as placing a unique demand on South African youth, which they indicated as a heavy burden and an isolating experience.

Moreover, the conversation regarding the unity of Africa, the diversity of Africa, how people are the same and different, and holding different expectations and realities based on the different colonial pasts in different African countries, or the effect of apartheid in different provinces in South Africa, surfaced. This also led to reminders of those who had died for freedom. Death was associated with skeletons, and skeletons in the closet led to a conversation about secrets held by elders that enable or inhibit progress in South Africa or African unity in general.

Even in writing this article, the authors were aware of having trouble keeping their eyes open. They drank more coffee and sought medical advice, finally concluding that the affect embedded in the article and the study is depressing, and as one reads and writes about it, one feels sad and funerial in relation to the people who have suffered and died through apartheid.

The setting of the dream was a dark room. Through association, this setting was linked to loadshedding in South Africa, which led to a conversation about privilege and access to resources and relating to students based on their socio-economic backgrounds and the role of student leadership pertaining to this. As a result, student leaders thought about South Africa during apartheid and after, including the role of the #Fallist movements in decolonization and transformation. The above has been characterized as feelings related to trauma related to the South African legacy. This feeling resonated, given the intense emotionality involved, the sadness in the room, heaviness or feeling drained, talks about anxiety, and explicitly disturbing examples of violence and injustice discussed.

**Figure 2:**
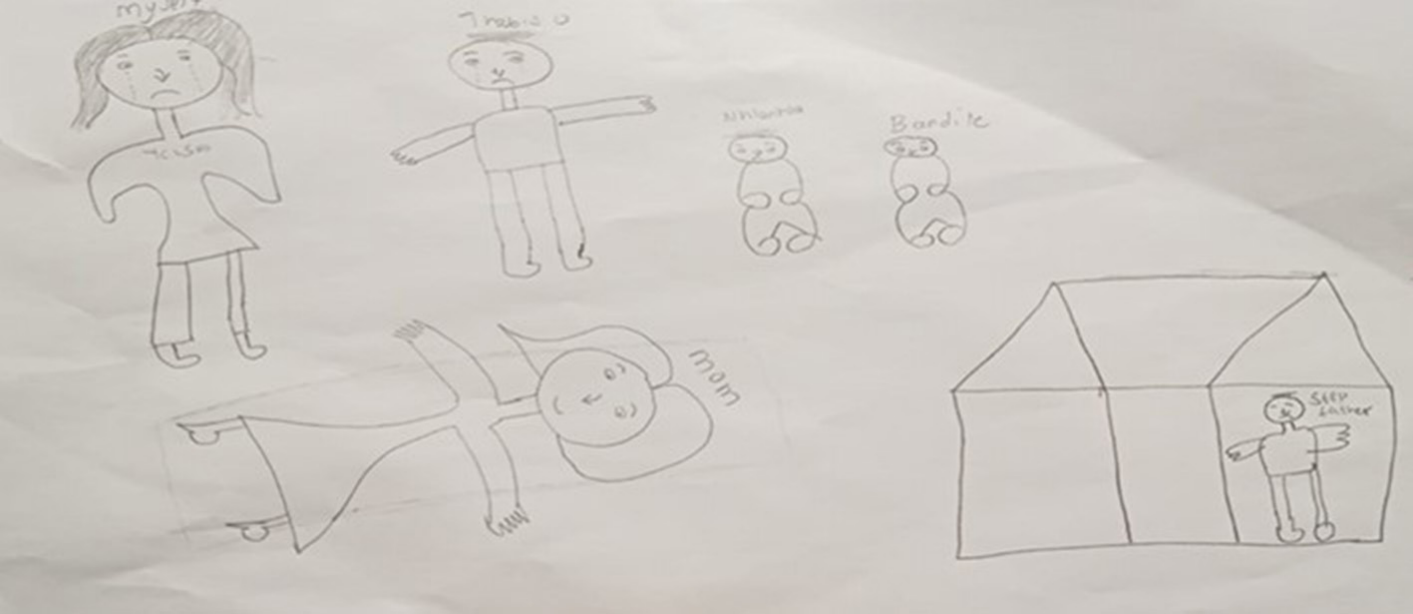
Dream drawing on absent fathers, sick mothers, and resented leadership.

In another dream drawing (Figure 2), the student leader shared a dream about the complexities of leadership. In this dream, she, her stepsiblings, and her mother were outside their house. Her mother was sick, almost dying. The dreamer took responsibility for nursing her mother back to health but felt resentment toward her biological (elder) brother, who was missing from the drawing. In addition, she felt resentment toward her stepfather, who locked himself inside the house alone for self-protection.

This drawing evoked a conversation about absent fathers and the frustration experienced by student leaders for missing leadership examples and having no one to look up to. The aftermath of history and politics that has resulted in rare examples of leadership by women, as well as the death of Mandela, who, in their words, left no one to look up to, advances into psychological and social impact. At the end of this conversation, the student leaders called social dream drawing a wound-healing session where they connected and learned about each other at a deep level for the first time, even though they had been student leaders at the same university for some time. This speaks to a desire and means for connection and how social dream drawing was related to enhanced collaboration and relationships, including a deep connection linked to sharing deep emotions by listening to each other in ways that they had not experienced before. Fake, step or *de facto* fathers were also depicted as letting the family face the brunt of the violence of apartheid behaviors.

**Figure 3:**
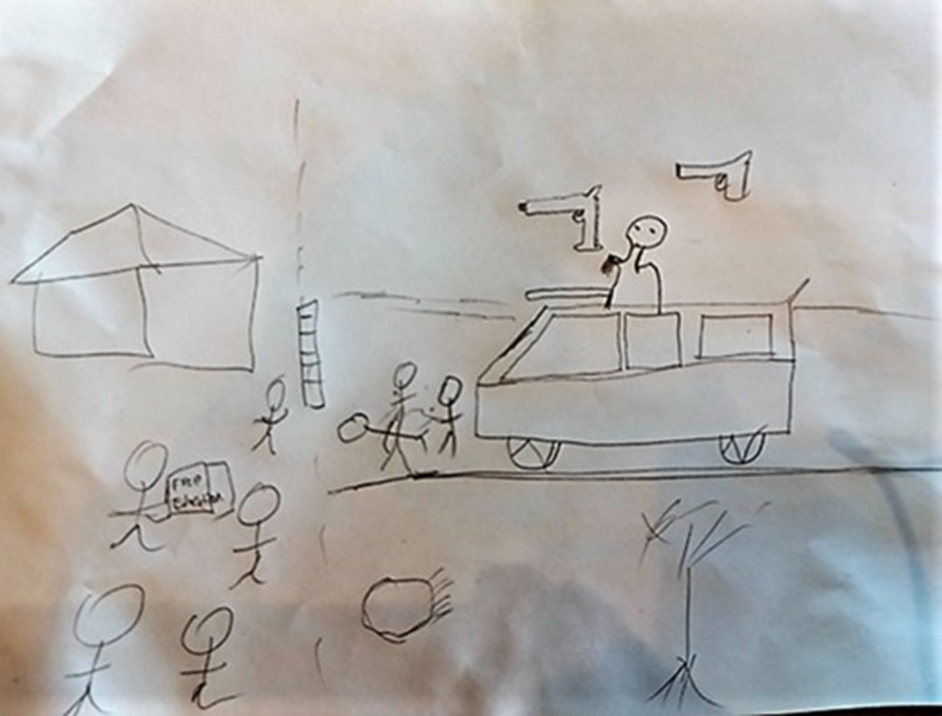
Dream drawing about protest and bondage in student leadership.

The last drawing (Figure 3) that we will include is one of many regarding dreams about protests (or organizing a protest or being part of a protest).

In this drawing, the student leader described an instance where he was part of a protest that he helped organize. At the university where he exercises student leadership, it has become difficult these days to organize a protest. In this case, in the dream that he was explaining, he (and his group) had a successful attempt. During the protest, he stood at the top of a massive van that resembled an army or military truck, shooting at a building of the university, although the gun was not in his hand but suspended from his hand. Some students were also shot at while protesting for free education.

The dream brought up a conversation about the contentious barriers relating to student leadership. These relate to the catch-22 about the legislative involvement of student leaders in university management and decision-making that stands in tension with the formal and informal demands of being a student, as well as the tightrope that student leaders walk in implementing their student leadership role without jeopardizing the attainment of their university degrees. Feelings of intimidation and frustration and feeling defeated and muted flared up. At an associative level, shooting at the building of the university was about attacking the institution in response to student leaders feeling silenced, thus expressing their frustration and inflicting impotence. The building is a monument, representative of a past they struggle to change. Here too, student leaders applied listening to each other in a way different from their other engagements. In their words, they appreciated how psychology facilitated a space to process and express difficult politics within the student leadership context. They felt bonded after social dream drawing.

At the same time, the conversation about bonding related to a social dream drawing at another university. This conversation focussed on the sense of bondage experienced in student leadership. Student leaders, at times, feel trapped within the system in terms of the catch-22 expressed above. They feel conflicted in their loyalties to their different group affiliations: the university that pays them, the students who elected them and whom they serve, the families they represent, and the political parties to which they belong. Psychoanalytically, this relates to a parallel process in the field that points to an associative unconsciously (Long, 2017) derived connection that social dream drawing facilitated.

## Discussion

The social dream drawing reveals the significance of unity (in student leadership, South African higher education, South Africa, and African leaders), themes about bondage (as an act of being close in relatedness, or bonded to one another, and simultaneously as restriction or slavery) and the emotional burden of student leadership contained in the metaphor of incapacitated or absent fathers, and elder daughters who step into leadership because of “sick” mothers.

In addition to the capacity of social dream drawing to drive compassion in itself, due to its participatory and engaging nature, using abductive reasoning for theory-building about student leadership role experience results in the emergence of compassion in terms of self-compassion (from the student leaders toward themselves), compassion toward one another, and researchers' compassion toward the student leaders. Accordingly, social dream drawing helped student leaders to express themselves, make sense of their experiences, and to process them—they felt the issue (emotionally or psychologically) and brought it to life through the drawing. Once that happened, they became sharper and better advocates. By hypothesis, this proto-mental level of communication is where we feel we connect authentically as embodied beings—alive animals—and from where we feel compassion emerge. Thus, social dream drawing acts as a mechanism to access information regarding the role experience of student leaders, opening space for a different kind of listening.

Trotter (1909) introduced the concept of herd instinct in the early 1900s (Swanson, 2013). In this, the inherent capacity for compassion was referred to. According to Trotter (1909), humans have the disposition to act in the interest of the social group; thus, he proposed a model of cooperation as a basis for adaptation. Suttie (1960 [1935]) followed by referring to our innate need for companionship. It was based on Trotter (1909) and Suttie (1960 [1935]), for instance, that Bion (1961, 1970), the British group psychoanalyst, developed his group work approach. According to Bion, groups are more powerful for health and healing. His “container-contained” concept (Bion, 1970) introduced “reverie” (Ogden, 1979; Katz, 2017) and the capacity of the mother to emotionally digest overwhelming feelings leaving the child feeling understood and contained. This transformed Klein's Projective Identification from an evacuative defense to a basis for emotional communication, including introjective identification (Ogden, 2004). Through reverie, there is an engagement that is achieved cognitively but also emotionally by an interception of rational functioning and receptivity toward listening without memory or desire - a means to identify with the other that creates a ‘new' we-ness or me-ness, in terms of Bion's Basic Assumptions of Group Dynamics (Bion, 1970). So, Bion, 1948, p. 20) indicated that mental life extends beyond the physical boundaries of the individual “transindividual”; hence, the relative indistinction between psyche and soma within the individual may in some way be correlated with the background of a substantial (relative) absence of a distinction between individuals. The field (in this case, the drawings and the dream) is thus the unconscious interaction being born for intersubjective determinants to be identified.

These primitive proto-mental elements being stirred or triggered by the leadership experience and tapping into dreams requires an emotionally containing environment that allows individuals and the group to understand and process the impact of these discoveries and changes occurring. The anxiety-inducing, politically subversive, or counterculture elements surfacing in social dream drawing require a psychologically safe space to engage with and unburden them, so that they are no longer suppressed or expressed in a subversive or aggressive manner (Winnicott, 1964; Bion, 1970; Long, 2002). To do this in a group context, student leaders need to be able to open up to one another in a shared common space, referred to psychoanalytically as “mutuality” (Benjamin, 2004; Harding, 2006). Rather than passive-aggressively complying (twoness) “me/you”, “us/them”, which is the usual university attitude, social dreaming and shared associations and drawings shift them to mutually engaging experiences, giving over to the other mutually, creating a third space for “me, you and us.” This requires each party to understand the other. It is in the process of mutual recognition that the possibility of experiencing the other in reality exists. In the complementary structures of the master-slave discourse fantasy, the other replaces the actual other. There is no struggle for recognition; instead, there is domination, idealization, submission, or related defenses (Harding, 2006).

The above-mentioned leads to the notion of Benjamin (2004) psychoanalytic third, which is how we get to go beyond the experience of the ‘doer, done to' dynamic in which we are either victim or persecutor. Harding (2006) pointed out that “surrendering yourself to the other's view, by empathic listening, compassion, and companionship, creates mutuality through the felt recognition and acknowledgment by the other”. Benjamin constructed a three-step process of identification, surrender, and gratitude (Benjamin, 2017, p. 204). According to her, recognition is a form of relational love that enables the stepping out of the “doer, done to” mentality into complementarity (Benjamin, 2011, p. 21).

These all relate to the relational philosophy that is mostly used in explaining African ontology. Ekanem (2012) suggested that African philosophy is about the seeking of meaning and understanding within the bias and context of African cultural setting and experience, where African philosophy is the reflection of an African, or those who are not African, on how Africans make sense of their existence and the world in which they live, based on the African cultural experience and reality. In this, ideas regarding Ubuntu emerge. Ubuntu premises that a person is a person (or human) because of others (Hailey, 2008). This working model or African philosophy predicts a relational paradigm of interconnectedness and interdependence that is rooted in solidarity among humans toward the achievement of reconciliation and co-existence (Ngubane and Makua, 2021). To make sense of this, Ubuntu is possible in contexts of compassion-companionship where strings of empathetic listening are strung.

We, therefore, propose that people are all connected associatively through the field like mushrooms. The root system of the plants, such as mushrooms (or fungi), called the mycelium, penetrates the roots of plants forming relationships and working together to increase resilience in the whole community (Stamets, 2005). As a great connector of the forest, mushrooms demonstrate one-ness, continuity, communication in relationships, and closeness (Spacal, 2017). Similarly, when learning emotionally, through compassion and companionship using empathetic listening, one starts to have images of what is happening with the other, surrendering self to the other. In this, student leaders can join together and be in a dream state with one another and be able to associate. The sharing of dreams, therefore, creates the field as such, resembling a social mycelium.

It is known that social dreaming is not conventionally viewed as therapy (Manley, 2014). Nevertheless, student leaders in this research referred to sharing dreams as wound healing. This sentiment was expressed in the following quotation:

Quotation 1

“*So, in wound-healing sessions like this where we bond and we learn about each other's dreams and we learn about what each of us has inside of them, yes, we do go through a lot all of us and it felt nice for once to be saying [it is not only happening to me - paraphrase]*.

In our interpretation as authors, the social dream-drawing process followed in this research unlocked compassion that allowed the group to process their experiences toward emotional growth and an integrated self and group that ultimately promotes positive social outcomes (Bion, 1961; Akhtar, 2013; Long, 2016). This emotional growth and integration of self and group occur instead of picking up unconscious messages such as shame, guilt, and anger, among other possible messages that can be enabled by the past of apartheid and colonization in South Africa. References regarding picking up these emotional messages are expressed in the following quotations:

Quotation 2

“*You are not going to be a good leader if you don't get angry. Because anger is the fuel of the revolution…….”*

Quotation 3

“*The pressure on the inside becomes too much that you become angry even if someone says a simple word, but you because you have already - you are traumatized! - it releases a sense of anger wherein interpersonal conflict that you can't control anymore.”*

The dreaming, sharing, and drawing built an empathic community that developed meaningful ways of processing these potentially vengeful and destructive feelings, transforming them into the community glue of self-other-self identification. This extended the individual's sense of self-boundaries and created a community of shared identification, meaning, and compassion for those who have gone before. This new generation of leaders, akin to the mushroom network of leaders, metaphorically linked arms to produce a resilient, living container to detox racist and apartheid hate and violence from the children of the traumatized victims. By connecting these ideas to the mushroom metaphor, we hypothesize that the use of social dream drawing with student leaders in South African universities calls for companionate (compassion and companionship) listening that drives emotional integration of self and group for restoration and creativity toward new and fresh thoughts and ideas about the student leadership experience in a South African university.

## Conclusion

The student leaders' reflection on social dream drawing involves the realization of the power of social dream drawing. This realization pertains to how it indeed drives compassion and facilitates the feeling of relatedness and connection for student leaders. In this, student leaders feel comfortable to the extent of the research pertaining to the idea that dreams allow the human capacity for connections that open space for compassion. Thus, social dream drawing provides an innovative and inclusive research agenda into new horizons of forms of compassion necessary within South African student leadership.

## Data availability statement

The original contributions presented in the study are included in the article/supplementary material, further inquiries can be directed to the corresponding author.

## Ethics statement

The studies involving human participants were reviewed and approved by the University of the Free State, General and Human Research Ethics Committee (GHREC). Additionally, institutional permission to conduct research at the various institutions was obtained through the respective institutions ethics committees. The participants provided their written informed consent to participate in this study.

## Author contributions

NP conceptualized the research, collected the data, and conducted the thematic analysis. Both authors contributed to the article and approved the submitted version.
